# Use of iRNA in the post-transcriptional gene silencing of necrosis-inducing* Phytophthor*a protein 1(NPP1) in *Phytophthora cinnamomi*

**DOI:** 10.1007/s11033-023-08562-7

**Published:** 2023-06-16

**Authors:** Patrick Pascoal-Ferreira, Abdessalem Chahed, Rodrigo Costa, Iuliia Branco, Altino Choupina

**Affiliations:** 1 Centro de Investigação de Montanha (CIMO), Instituto Politécnico de Bragança, Campus de Santa Apolónia, 5300-253 Bragança, Portugal; 2 Laboratory for Research on Genetics Biodiversity and Bioresources Valuation of (LR11ES41), ISBM, University of Monastir, 5000 Monastir, Tunisia

**Keywords:** *Phytophthta cinnamomi*, *Castanea sativa*, iRNA, Hairpin-RNA, NPP1

## Abstract

**Background:**

*Phytophthora cinnamomi* is an Oomycetes associated with soil, this Oomycete is one of the most destructive species of *Phytophthora*, being responsible for the decline of more than 5000 ornamental, forest, or fruit plants. It can secrete a class of protein NPP1 (Phytophthora necrosis inducing protein 1), responsible for inducing necrosis in leaves and roots of plants, leading to their death.

**Objective:**

This work will report the characterization of the *Phytophthora cinnamomi* NPP1 gene responsible for the infection of *Castanea* *sativa* roots and will characterize the mechanisms of interaction between *Phytophthora cinnamomi* and *Castanea sativa*, by gene silencing NPP1 from *Phytophthora cinnamomi* mediated by RNAi.

**Methods and results:**

For silencing a part of the coding region of the NPP1 gene, was placed in the sense and antisense directions between an intron and ligated to the integrative vector pTH210. Cassette integration was confirmed by PCR and sequencing on the hygromycin-resistant *Phytophthora cinnamomi* transformants. Transformants obtained with the silenced gene was used to infect *Castanea sativa*.

**Conclusions:**

Plants infected with these transformants showed a great reduction in disease symptoms, confirming iRNA as a potential alternative biological tool in the study of molecular factors, and in the control and management of *Phytophthora cinnamomi.*

**Supplementary information:**

The online version contains supplementary material available at 10.1007/s11033-023-08562-7.

## Introduction

### *Phytophthoracinnamomi*

*Phytophthora cinnamomi* resides in the soil and attacks fine roots, infecting only healthy tissue from which it extracts the nutrients it needs. The species is native to Southeast Asia and has spread globally through plant exports to Australia, Europe, North America, and South Africa. Today, over 5000 host plants, including ornamental, forest, and fruit varieties, are known to be susceptible to this pathogen [1, 2]. Recently, it has been ranked as one of the top 10 plant pathogenic Oomycetes of agricultural and scientific importance [3, 4].

In Portugal, *P. cinnamomi* is responsible for considerable declines in chestnut (*Castanea sativa Mill*), cork oak (*Quercus suber L*.), and holm oak (*Quercus rotundilolia Lam*) populations, resulting in significant economic losses and damage to the natural ecosystem and cultural heritage of affected regions [5, 6]. Indeed, recent literature suggests that few oomycetes cause as much destruction as *Phytophthora cinnamomi*, which is responsible for rotting the roots of countless host plants.

*Phytophthora cinnamomi* can survive for extended periods in soils and sporulate when favorable conditions arise, producing numerous biflagellated zoospores [7]. These mobile zoospores move to appropriate locations to infect and spread throughout the plant [8]. Moisture is a potent driver of the fixation, dissemination, and longevity of *Phytophthora cinnamomi*; this oomycete can survive for up to 6 years in humid soils and relies on moisture for asexual reproduction via sporulation. Moisture also contributes to the formation and release of sporangia and aids in the activity of mobile zoospores [9-11].

The mycelium, oospores, or chlamydospores can survive in the soil or, in the absence of a host, they can remain in dead plant tissue. Germination occurs when there is a certain amount of moisture in the soil, and this process is responsible for producing sporangia at temperatures between 18 and 30 °C [12]. Sporangia, in turn, germinate directly or indirectly and form and release zoospores, which are one of the main factors of *Phytophthora* infection [2, 13].

### Molecular effectors of plant-associated pathogens

The term “effector” has been widely used in studies of plant-pathogen interactions [14]. Besides being identified in phytopathogenic microorganisms, effectors have recently been identified in plant pests, such as aphids [15], and in mutualistic fungi, such as *Laccaria bicolor* [16], *Glomus intraradices* [17], and *Piriformospora indica* [18]. Consequently, effectors are now defined as molecules secreted by plant-associated organisms that modify the structure and function of the host cell [19].

Several pathogens, including fungi and oomycetes, colonize the intercellular spaces of plants and secrete effectors that target plant defense molecules by acting on the apoplast [20]. Classic examples include the *Cladosporium fulvum* effectors Avr2, Avr4, and Ecp6. During infection, Avr2 inhibits at least four tomato cysteine proteases, which contributes to the virulence of the pathogen, as these proteases are important components of host defense [21].

Different sets of effectors are secreted at different stages of parasitism [22]. The fungi *Colletrotrichum higginsianum* and *Colletotrichum graminicola* infect Arabidopsis and maize, respectively. These pathogens use transcriptional regulation to synthesize and secrete different sets of effectors and enzymes important at different stages of infection. For example, the biotrophic phase is characterized by an increase in the expression of effectors and secondary metabolism enzymes, while in the necrotrophic phase, there is an increase in the expression of hydrolases and transporters [23]. In *C. higginsianum*, regulation is most refined during the pre-penetration stage, where candidate effector genes are upregulated to prepare for entry into the host cell [22].

In *Phytophthora spp*., several RxLR-type effectors are induced during cyst germination or in the early stages of infection, while several Nep1-like proteins, which induce necrosis, are expressed in advanced stages of infection, indicating that these proteins contribute to the necrotrophic phase of pathogenesis [24, 25]. The *Phytophthora infestans* SNE1 effector is transcriptionally upregulated during the biotrophic phase, supposedly to keep the host cell alive by suppressing the action of cell death inducers that are expressed during the necrotrophic phase of the interaction [26]. Since the expression of effectors occurs in a regulated manner at defined times and places during the interaction with the host, they can interact with plant proteins to exert their functions, promoting a successful infection of the phytopathogen [14, 20, 25, 27].

### NPP1 protein in *Phytophthoracinnamomi*

Pathogens of various taxonomic origins, including bacteria, fungi, and oomycetes, have been shown to secrete and inject molecular effectors into the apoplast and cellular cytoplasm of plants to establish infection and suppress host defenses [14, 28, 29]. The necrosis-inducing protein NPP1 (Necrosis-Inducing *Phytophthora* Protein 1), is secreted by virtually all *Phytophthora* species, but it was only recently identified as belonging to the NEP-like protein class (NEP Like Proteins) [30]. The main function of NLPs is not yet sufficiently clear. However, it is known that this class of protein operates as a determinant factor for virulence, aiding and potentiating the growth of diseases in host plants [31, 32].

The NPP1 protein produced by *Phytophthora cinnamomi* is thought to cause necrosis in the leaves and roots of the plant, leading to plant death during the necrotic phase [14, 33, 34]. Before dying, plant cells manifest a hyper-defensive state that leads to a series of mechanisms, including the release of ethylene, an increase in the activity of kinase enzymes, production of phytoalexins, induction of the PR transcription gene, an increase in cytoplasmic levels of Ca^2+,^ and induction of rapid changes in physiology and gene expression. All these mechanisms are enabled by NPP1 [14, 32].

Therefore, studying the factors that affect the expression of NPP1 is extremely important to better understand its role in the infection mechanism of *P. cinnamomi* in plants such as *Castanea sativa*.

### RNAi for genetic improvement of cultivated plants

To improve the quality of cultivated plants, the RNAi technique has been utilized to make them more resistant to pests [33]. For instance, in barley, the RNAi technique has been successfully employed to confer resistance against yellow dwarf virus [35]. In rice plants, it has been utilized to reduce the level of glutenin, resulting in a type of rice with a lower protein content that is beneficial for patients with protein-related health issues [34, 36].

Moreover, this technique can be employed to produce plants with lower levels of toxins [33]. For instance, cottonseeds contain dietary protein, but they also contain a protein that is toxic to humans. Therefore, RNAi was used to generate cotton plants whose seeds had reduced levels of the enzyme required for the synthesis of this toxic protein [37].

## Materials and methods

### Biological materials

#### *Phytophthoracinnamomi* isolates

The pathogen *Phytophthora cinnamomi* used in this study was obtained from soil samples collected in northern Trás-os-Montes, Portugal, from chestnut trees (*Castanea sativa Mill*) that were infected by this oomycete. The isolate was identified and maintained in the *Phytophthora* collection of Escola Superior Agrária de Bragança, Portugal, with identification label-Pr120.

#### *Escherichiacoli* DH5α

To prepare the genetic constructions, in this work, we used the prokaryotic strain of *Escherichia coli* DH5α, of genotype (supE44, ∆lacU169, (f80lacz∆M15), hsd R17, recA1, endA1, gyrA96, thi-1, relA1).

#### *Castanea* s*ativa*

The micropropagation of adult clones of chestnut plants was carried out according to the method described by Martins [38]. The method consists of inoculating the axillary buds, multiplying the meristems by axillary sprouting, and elongating the shoots. *Castanea sativa* seeds were sterilized and germinated in sterile vermiculite and then grown in a greenhouse until their root length reached 5–10 cm or 25–30 cm [38].

### Culture media

All microorganisms used in this study were cultured in autoclaved media at 120 °C for 20 min and prepared in ultrafiltered (Milli®) and double-distilled water. All percentages are given in a weight/volume ratio. Solid media were prepared by adding 2% agar to the corresponding liquid media. For selection of clones for antibiotic resistance, media were supplemented with ampicillin (50 µg/mL) and kanamycin (50–100 µg/mL). When necessary, several antibiotics were added simultaneously, always just before pouring the media onto the plates. Reagents from usual suppliers with quality guarantees (Merck, Sigma, etc.) were used unless otherwise mentioned.

#### Culture medium used for *Escherichiacoli*

*Escherichia coli* growth was carried out in solid medium (on Petri dishes) or in liquid medium (in Erlenmeyer flasks or in test tubes). Cultures in liquid medium were carried out with agitation at 250 rpm, ensuring that the volume of the culture medium did not exceed one third of the volume of the container. Bacterial growth was monitored by measuring absorbance at a wavelength of 600 nm and counting cells in a Thoma chamber. Bacterial cultures were grown in Luria Bertani (LB) or YEPD (Yeast Extract Peptone Dextrose Medium) with aeration at 37 °C and 180 rpm on an orbital shaker. Antibiotics were added to the culture medium according to the resistance presented by the plasmids used.

#### Growth condition for *Phytophthoracinnamomi*

##### V8 liquid medium

V8 medium was prepared by adding to 330 mL of V8 juice (Campbell Soup Co, Camden, NJ), 4.5 g of calcium carbonate (CaCO_3_) and stirred for 30 min. Culture media was transferred to 1000 mL centrifuge flasks and centrifuged at 2500×*g* for 15 min at 20 °C. The supernatant was then, poured into a new flask without disturbing the pellet. The cleared V8 juice (V8 broth) was then, diluted 10-fold with ultra-filtered water and autoclaved at 120 °C for 20 min.

##### V8 solid medium

It was prepared as aforementioned, but 15 g of agar per 1000 mL was added to the diluted V8 broth before autoclaving. After sterilization, the culture media was poured into Petri dishes, allowed to solidify at room temperature and kept at 4 °C.

##### YEPD (yeast extract peptone dextrose medium)

The liquid YEPD medium was prepared by adding the following: yeast extract 1%, peptone 2% and d-glucose 2%. The solid YEPD medium was prepared as above but adding 2.0% Agar.

##### PDA medium

PDA (Potato Dextrose Agar—HIMEDIA) was prepared by adding 39 g of commercial PDA powder to 1 L of ultra-filtered water, then autoclaved at 120 °C for 20 min.

### Maintenance and conservation of microorganisms and plasmids

The mycelium of the filamentous microorganism, *Phytophthora cinnamomi*, was periodically subculture into Petri dishes with solid PDA or V8 and stored long-term in 30% glycerol (V/V) in water at a temperature of − 20 °C.

The bacterial strain used in this work was stored at − 80 °C in 2 mL cryotubes, in the respective culture medium with 1/3 of the volume of 100% glycerol. When using it, inoculations were carried out in the Petri dish with Luria Bertani LB-agar medium and stored at 4 °C.

Plasmids after being properly purified were stored for short term at 4 °C and for long term they were stored at − 20 °C.

### Expression vectors

More information about expression vectors used, consult the supplementary material.

### DNA extraction

For the extraction of *Phytophthora cinnamomi*’s genomic DNA, the oomycete was grown in PDA medium, covered with aluminum foil at 25 °C and after 6 days of mycelial growth, DNA was extracted. The process of extraction consisted in the use of a Lysis Solution, 200 mM Tris-HCL; 25 mM EDTA; 250 mM NaCl and SDS 0.5% (w/v) followed by a deproteinisation with phenol/chloroform/isoamyl alcohol (25:24:1) and precipitation of DNA by washing with ethanol (100–70%) at − 20 °C. The DNA pellet was then dissolved in ultra-filtered water. The treatment of DNA was done with RNase 5 mg/mL for 5 min at 37 °C.

#### Visualisation and purification of nucleic acids in agarose gels

The visualization and separation of DNA fragments were performed by electrophoresis in agarose gel low melting 0.8% (w/v) in TAE (Tris-Acetate 40 mM, 1 mM of EDTA), with 0.5 µg/mL GreenSafe Premium (NZYtech,Portugal), for 40 min at room temperature, at 80 V.

After irradiation of the gel using the ChemiDoc™ XRS + imaging system (BioRad) it was possible to view the size and intensity of the desired band by comparison with DNA ladders.

The isolation and purification of DNA fragments from agarose gel were made by cutting the agarose band containing the desired fragment from gel prepared in TAE with a clean razor blade. DNA molecules present in the band were purified by the QIAquick® Gel Extraction Kit (QIAGEN, Germany) following the manufacturer’s instructions.

### SenseIantisNPP1 silencer cassette construction

#### Design of silencing cassette (shRNA based vector) for NPP1 gene

Several methods are used for constructing silencing cassettes: including annealed oligonucleotide and PCR-based cloning. The PCR-based method has been chosen because it is cheaper and reliable. The silencing shRNA construct was made by joining a 5′sequence fragment to another fragment from the 3′ end of the same sequence in an inverted orientation (by performing two separate PCR reactions) separated by a spacer or loop DNA. Transcripts generated from such constructs will have regions of self-complementarity that have the potential to form shRNA duplexes that generate a siRNA in the cell capable of degrading specific sequences of mRNA.

The amplification of the sense sequence selected within the ORF of *NPP1* gene has been done using specific primers designed following Tiscornia et al. protocol (Table 1) [39].

**Table 1:** List of primers used for *NPP1 *silencing cassette construction

Name	Sequence	Tm °C	GC %
FPCR1	5′GATAGGGCCCATGTGCACGACATGTTTACT3′	65.6	50
RPCR1	5′GCGACGGTGAAGACAACCGT3′	62.4	60
FPCR2	5′GCGACGGTGAAGACAACCGT3′	62.4	60
RPCR2	5′CTAGGGGCCCAAAAAAATGTGCACGACATGTTTACT3′	66.4	44.44

The PCR product was purified following the protocol of the commercial Wizard® SV PCR Clean-Up System (Promega, USA), which consists in passing the sample through a chromatography column, in which, the nucleic acids together with a membrane binding solution are retained on the silica membrane then the components of PCR mix are eliminated. Finally, the DNA is eluted in a nuclease free water.

The ligation to the loop sequence (provided by Professor *Sophien Kamoun—*The Sainsbury Laboratory (TSL), Norwich, UK) was done using T4 DNA Ligase (Promega) and following the manufacturer’s instructions.

For the amplification of the antisense sequence a forward primer with approximately 20 nucleotides and a reverse primer including: (1) 4–5 extra nucleotides to assist in digestion; (2) 3′ restriction site recognition sequence; (3) AAAAA termination sequence complementary strand; and (4) 20 nucleotides complementary of 3′ antisense strand sequence were used. The PCR product was purified as aforementioned and ligated with T4 DNA Ligase (Promega) to the fragment containing the sense sequence and the loop.

The construct with approximately 551 bp length, was purified after ligation and then digested with *Apa*I restriction enzyme in a final volume of 30 µL with a reaction buffer (1×) (Promega), 1.5 µL (7U/microl) of *Apa*I enzyme, 1 µg/µL DNA, for 4 h at a temperature of 37 °C.

The digested construct was then visualized on a 1% agarose gel, and the 500 bp band of the DNA excised with a clean razor blade and purified using the QIAquick® Gel Extraction Kit according to manufacturer’s instructions (QIAGEN, Germany) so that can be ligated directly by T4 DNA ligase (Promega) to the pUC57 vector digested previously by *Apa*I restriction enzyme.

### Extraction of the plasmid DNA of the transformed *Escherichiacoli*

The bacterial colonies, that have grown during the incubation period and which were expected to contain recombinant plasmids, have been picked off and transferred to the LB liquid media containing 100 µg/mL of ampicillin antibiotic. The cultures were incubated overnight on the shaker at a shaking rate of 200 rpm at 37 °C.

The plasmid miniprep has been made, using the NZYMiniprep kit (NZYtech, Portugal) according to manufacturer’s instructions which is based on alkaline lysis of bacterial cells followed by adsorption of DNA onto silica. Afterward, the recombinant plasmid was digested by *Apa*I restriction enzyme then, the digested products were subjected to electrophoresis in agarose gel. The insert was purified from the gel by QIAquick® Gel Extraction Kit according to manufacturer’s instructions (QIAGEN, Germany).

### Cloning the silencing cassette into the pTH210 vector

The pTH210 vector was extracted from *Escherichia coli* previously transformed, digested with ApaI enzyme; as described for the cassette digestion and then dephosphorylated with Calf Intestinal Alkaline Phosphatase (CIAP).

Dephosphorylation was performed to remove phosphate groups from the 5 ends of the linearized plasmid and prevent recircularization. The linearized plasmid was incubated with CIAP, “Calf Intestine Alkaline Phosphatase” (Promega, USA) (0.01 u/µL) with 5 µL of the appropriate buffer CIAP 10× and ultra-filtered water to a final volume of 50 µL for 30 min at 37 °C.

The fragments of the silencing cassette and the vector pTH210 were excised with a clean razor blade and purified using QIAquick® Gel Extraction Kit according to manufacturer’s instructions (QIAGEN, Germany) then ligated using T4 DNA Ligase enzyme.

To clone the cassette into the pTH210 vector the ligation of DNA fragments was carried out in a 10 µL standard ligation reaction. The reaction contained DNA fragments with compatible ends; 100 ng of plasmid, 0.5 µL of T4 DNA ligase, and a 1 µL of ligation mix buffer (Promega). Reactions were incubated at 4 °C overnight.

The recombinant vector was used to transform *E. coli* NZY5 α competent cells. After plasmid extraction, digestion with restriction enzymes, was performed to confirm the cloning of the cassette into the pTH210 vector.

For more information about Cloning the silencing cassette into the pTH210 vector, consult the supplementary material.

### Genomic analysis of the transformed *Phytophthoracinnamomi* (PCR & DNA sequencing)

The mycelium of transformed *Phytophthora cinnamomi* was selected in order to extract the genomic DNA and make a screening PCR to confirm the integration of the pTH210 into *P. cinnamomi* genomic DNA. The screening PCR was performed with the primers listed in Table 1.

Amplification products were checked by agarose gel electrophoresis and samples were sent to the Department of Microbiology and Genetics, University of Salamanca for sequencing with respectives primers and using an automated sequencer ABI PRISM 377 W that performs the electrophoretic separation and detection of DNA fragments labeled with fluorescent markers.

### *Castaneasativa* infection with *Phytophthoracinnamomi* strains 

Infection of *Castanea sativa* roots was performed using mycelium of *Phytophthora cinnamomi* transformed and non-transformed to compare the effect of gene silencing on the plant phenotype. The roots were covered with fully colonized V8 medium, the plants were placed in sterile vermiculite and incubated for 72 h at 25 °C.

### Extraction of the plasmid DNA of the transformed *E*. *coli*

This task was done as described in "Extraction of the plasmid DNA of the transformed *Escherichia* *coli*" Section. After extraction, the recombinant plasmid with *NPP1* gene was digested by *Not*I and *Sac*II restriction enzymes, the digested products were subjected to electrophoresis in agarose gel.

### Protein analysis in SDS polyacrylamide gel—PAGE

The protein was analyzed on a polyacrylamine gel with SDS (SDS-PAGE), consisting of a concentration gel with 4% (w/v) of acrylamide and a gel of resolution with 15% of acrylamide (w/v).

Initially, the resolving gel was prepared consisting of 15% (w/v) acrylamide 1.5 M Tris-HCl to a final concentration of 25% (v/v). For gel polymerization, 10% APS (ammonium persulfate) was added to a final concentration of 0.05% (v/v) and TEMED (N, N, N′, N′-tetramethylethylenediamine) 0.05% (v/v).

The resolving gel was poured between two glass plates (components of the electrophoretic separation apparatus used Mini Protean 3, Biorad) and overlaid with a small volume of isopropanol until polymeriz. The concentrating gel was then poured, preparing it described above for the resolving gel, but replacing the 1.5 M Tris HCL solution with 0.5 M Tris and using twice the amount of TEMED. Then, an appropriate comb was placed to form wells in the gel for application of the samples. Before being placed on the gel, the samples were mixed with sample buffer (4% SDS (w/v), 50% glycerol (v/v), 0.25 M Tris HCL, 0.005% bromophenol blue (w/v) and 10% (v/v) B-Mercaptoethanol, followed by denaturation for 4 min at 95 °C.

## Discussion and results

### DNA extraction of *Phytophthoracinnamomi*

After six days of mycelial growth in the dark at 25 °C, the genomic DNA of *Phytophthora cinnamomi* was extracted to build up the *NPP1* silencing cassette and to amplify the ORF of the gene.

### Synthesis and preparation of the NPP1 silencing cassette

To synthesize an efficient conventional silencing cassette that targets the corresponding mRNA of the *NPP1* gene, several measures must be taken into consideration:

(1) The potential construct sequence should not have a perfect match of more than 16 nucleotides to an off-target gene of *Phytophthora* *cinnamomi* (verified by BLAST search) (http://www.ncbi.nlm.nih.gov/BLAST/). (2) The construct should not have an internal repeat or palindromes and must include low-to-medium G/C content. (3) A silencing cassette fragment between 300 and 1000 bp in length is recommended to maximize the efficiency of silencing obtained.

**Fig. 1:**
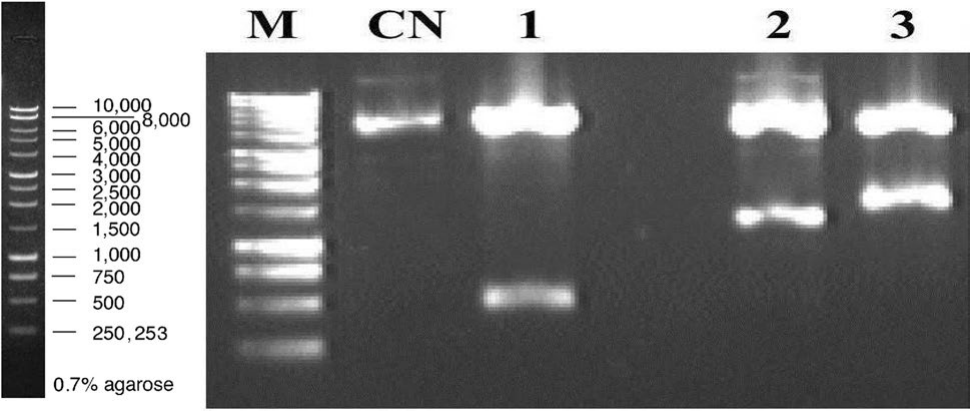
Enzymatic digestion of pTH210 with *Apa*I, *Sma*I and *Pst*I. 0.7% (w/v) agarose gel in 1X TAE. M) 1 kb molecular weight marker (Promega®); CN: Negative control for supercoiled non-recombinant pTH210 plasmid DNA; (1) *Apa*I digested recombinant pTH210 plasmid; (2) Recombinant plasmid pTH210 digested with *Sma*I; (3) Recombinant plasmid pTH210 digested with *Pst*I.

The sense and anti-sense sequences were produced by performing two separate PCR reactions, and then the PCR products were ligated by T4 DNA ligase. The generated cassette was purified, digested with *ApaI* enzyme, and then visualized on 1% agarose gel (Fig. 1).

The gel showed a specific band of 519 bp in length with good DNA quality, confirming the success of producing a silencing cassette that can trigger post-transcriptional gene silencing.

RNA interference using shRNA was chosen because previous studies have shown that the shRNA-expression system is more efficient than siRNA expression systems [40]. Additionally, the cost of shRNA construction is cheaper compared to synthesized siRNAs, and shRNAs are recommended for stable and enforceable expression as they integrate the genome and can be expressed for up to three years, while synthesized siRNAs are degraded after 48 h [41, 42].

### Transformation of *Escherichiacoli* DH5α with the NPP1 silencing cassette

The silencing cassette was purified using the QIAquick® Gel Extraction Kit and The silencing cassette was purified using the QIAquick® Gel Extraction Kit and cloned into the pUC57 vector for transformation into *Escherichia coli* DH5α competent cells. The transformed bacteria that were able to grow in medium containing ampicillin were selected for further experiments.

We chose to construct the cassette in the pUC57kan vector which has a favorable polylinker for this construction. After ligating the pUC57kan vector and the *senseIantisNPP1* cassette, *E. coli* DH5α was transformed with the recombinant DNA. After the selection of transformants in medium containing kanamycin, extraction and enzymatic digestion were performed.

To confirm the construction of the cassette in the pUC57Kan vector, an enzymatic digestion with *Apa*I was performed. Two bands were observed for the pUC57kan vector, one with a size of 2579 bp corresponding to the vector and another of 519 bp corresponding to the *senseIantisNPP1* cassette insert (see Fig. 1).

*Escherichia coli* cells transformed with the ampicillin-resistant pTH210 expression plasmid were also cultured, and the plasmid was extracted and then linearized using the *ApaI* enzyme, was observed. A 5549 bp band corresponding to plasmid pTH210 linearized by the enzyme *Apa*I. The products resulting from the enzymatic digestions were analyzed on an agarose gel, as shown in Fig. 1.

To ligate the *senseIantisNPP1* cassette in pTH210, the cassette was isolated after digestion with the *ApaI* enzyme. The fragments were separated on an agarose gel, cut from the gel, and purified using the gene clean kit. After purification of the pTH210 plasmid and the senseIantisNPP1 cassette, the cassette and the respective plasmid were ligated. The ligation product was used to transform competent *E. coli* DH5α cells. After plasmid extraction, the cloning was confirmed by enzymatic digestion with the enzymes *ApaI, SmaI, and PstI*, as shown in Fig. 1.

After digestions on an agarose gel, all the bands released by each of the enzymes were observed. As expected, in the enzymatic digestion with the *ApaI* enzyme, two bands were observed for the pTH210 vector, one with a size of 5030 bp and another with a size of 519 bp corresponding to the *senseIantisNPP1* cassette insert. Digestion with the SmaI enzyme released a larger fragment of size 4289 bp and a smaller one of 1260 bp, while the *PstI* enzyme released a larger fragment of 4115 bp and a smaller one of 1434 bp. These results confirmed the successful cloning of *senseIantisNPP1* in pTH210.

### Confirmation of *Phytophthoracinnamomi* transformation by culture in medium containing hygromycin antibiotic

The recombinant plasmid was used to transform *Phytophthora cinnamomi* by electroporation. The transformants were then cultivated on medium containing hygromycin (200 µg/mL) and incubated for 10 days at 25 °C in the dark. At the same time, non-transformed *P. cinnamomi* were incubated under the same conditions as negative control plates in medium containing hygromycin (200 µg/mL).

**Fig. 2:**
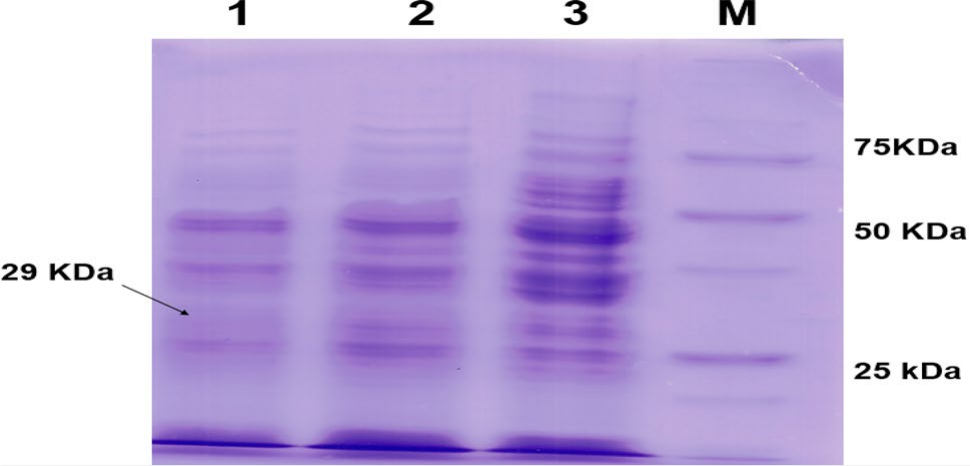
SDS-PAGE of expression in wild type/recombinant *Phytophthora cinnamomi* with 72 h of induction. (1) Absence of NPP1 protein expression in *P. cinnamomi* transformed with circular TE.8 supercoiled plasmid; (2) Partial expression in *P. cinnamomi* with the linear T1 plasmid; 3. Expression of NPP1 protein in wild type *P. cinnamomi*; M. 250 kDa protein marker

After ten days of incubation at 25 °C in the dark, there was no growth of the negative control in medium containing the hygromycin selection marker. Meanwhile, only six out of ten transformed *P. cinnamomi* grew in the medium with hygromycin (see Fig. 2). We can conclude that the transformation efficiency with electroporation was low, but better compared to the chemical transformation with PEG/CaCl_2_ [41].

The growth of transformed *P. cinnamomi* in medium containing hygromycin indicates that they can produce the hygromycin phosphotransferase (HPT) protein, which degrades the hygromycin and provides antibiotic resistance to these microorganisms. This transformation was only possible with the transformation of *P. cinnamomi* by the pTH210 vector, meaning that the transformation was successful.

No growth rate difference was observed between the wild type and the transgenic strains in the absence of antibiotic selection.

### Genotypic confirmation of *Phytophthoracinnamomi* transformation

After digestion in an agarose gel, all the bands expected to be each of the enzymes were observed. As expected in the enzymatic digestion with the *ApaI* enzyme, two bands were observed for the pTH210 vector, one with a size of 5030 bp and another with a size of 519 bp corresponding to the *senseIantisNPP1* cassette insert. Digestion with the SmaI enzyme released a fragment of size 4289 bp and another of 1260 bp. Meanwhile, the PstI enzyme released a fragment of 4115 bp and another of 1434 bp. These results confirmed the successful cloning of *senseIantisNPP1* in pTH210 (Fig. 1).

After PCR amplification, bands were produced with sizes of 565 bp for the hygromycin-cassette segment and 1090 bp for the hygromycin segment.

Enzymatic digestions of the two PCR products were carried out to further confirm their identities. After studying the restriction map of the two PCR products, the NcoI enzyme was used to digest the 565 bp fragment, cutting it at place 283 and generating two bands of 283 bp each. Digestion of the 1090 bp fragment was performed with the PstI enzyme, which cut at the 282pb place of this fragment and generated a band of 282 bp and another of 808 bp.

### Sequencing of the transformed *Phytophthoracinnamomi*

To confirm the integration of the recombinant pTH210 vector in the *Phytophthora cinnamomi* genome, the PCR products of the hygromycin fragment and the *senseIantisNPP1* silencing cassette were sequenced and analyzed with the BioEdit program. After analysis, the nucleotide sequences were blasted against the NCBI database to investigate the ID of the sequenced genes. The alignment of similar genes was analyzed and compared using the Muscle tool server (CLUSTAL multiple sequence alignment) from the EMBL-EBI database. The results of the alignment can be consulted in the supplementary material.

The alignment results of the hygromycin fragment sequence showed a high identity with the corresponding gene. However, for the NPP1 cassette sequence, only the beginning of the sequence corresponding to the sense strand of the silencing construct showed complete homology with the *P. cinnamomi NPP1* gene. This is because the construction of the cassette was based only on the sense sequence selected within the ORF of the *NPP1* gene sequence. The antisense sequence is inverted compared with the gene sequence, and the loop does not have any homology with the *NPP1* gene sequence. These results confirm that the silencing cassette has integrated into the genome of *P. cinnamomi.*

### Confirmation of transformation in *Phytophthoracinnamomi* through analysis of NPP1 protein expression on SDS-PAGE

To verify the effectiveness of the transgenic sequence *senseIantisNPP1* in silencing the endogenous gene *NPP1* in the transformed *Phytophthora cinnamomi* strain, protein production was analyzed in V8C liquid medium. This medium is considered suitable for the production and isolation of proteins related to this study [42].

After culturing the samples in the inducing medium, a negative control and samples from the transformants were collected after an induction period. To lyse the cells, Breaking Buffer lysis solution and 0.5 mm glass beads were used with vortexing, soluble proteins were extracted using SDS loading buffer (2×).

**Fig. 3:**
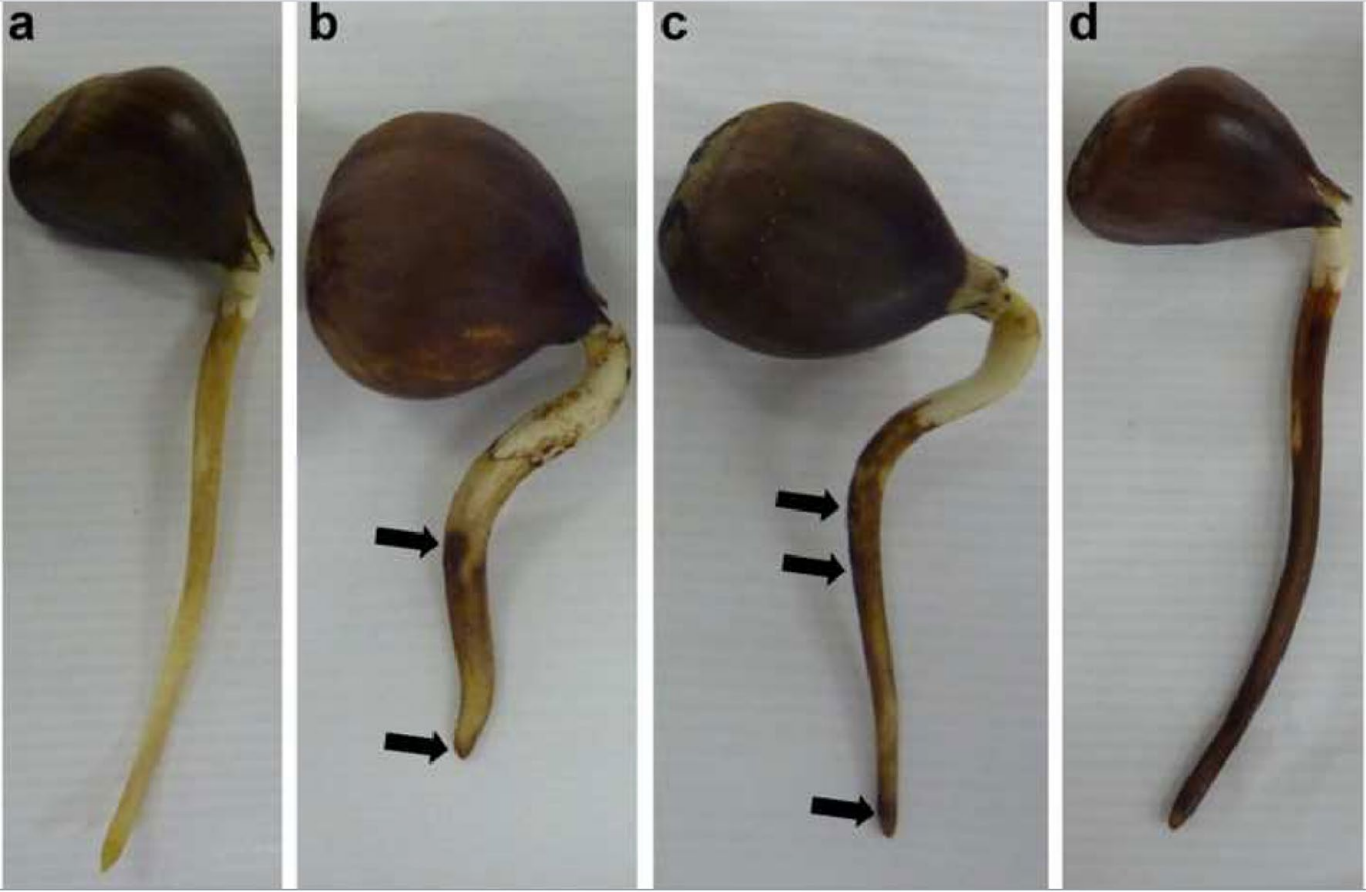
Morphological analysis of *Castanea sativa* roots. **A** Control of chestnut root not infected by *Phytophthora cinnamomi*. **B** Infected root after 12 h showing some necrosis. **C** Infected chestnut root after 24 h with intensified necrosis region. **D** Root infected by *P. cinnamomi* after 36 h showing almost entirely necrotic tissue

SDS-PAGE polyacrylamide gel electrophoresis was performed for wild type *P. cinnamomi* and two samples of transformed *P. cinnamomi* strains induced for 72 h (Linear T1, circular TE.8). It was expected that the wild type *P. cinnamomi* sample would show a band profile with a fragment corresponding to the Npp1 protein (estimated size 29 KDa), while the transformed strains with the *senseIantisNPP1* cassette were expected to have an absence of the corresponding protein fragment, as compared with the protein marker (Fig. 3).

Upon observing the results and comparing them to a protein marker, it was confirmed that the NPP1 protein was expressed in the transformed *P. cinnamomi* strain, but not in the circular TE.8 transformed strain, indicating successful silencing of the expression of the NPP1 protein. In well number 2, partial expression was observed for the strain transformed with the linear T1 plasmid, indicating a significant reduction in protein production compared to the wild type strain.

### Chestnut root infection with *Phytophthoracinnamomi* inoculum

The hyphae of *Phytophthora cinnamomi* can grow intracellularly in the root cortex of *Castanea sativa* by opening their way through the secretion of enzymes that degrade the host cell walls. In this intracellular growth, the pathogen penetrates the cell wall but remains surrounded by the host cell membrane, forming nucleated intracellular hyphae or non-nucleated intercellular hyphae. Plants reinforce cell walls at sites of contact with the pathogen, either through incompatible or incompatible interactions, regardless of other defense mechanisms [43]. The ease and speed with which the pathogen colonizes tissues determine its virulence, as it can avoid possible host defense responses.

For the infection assays, strips of V8Ca colonized with mycelium from wild type and transformed *P. cinnamomi* isolates were placed on roots that ranged from 5 to 30 cm in length and originated from *C. sativa* roots.

### Morphological analysis of chestnut root infection with wild type *Phytophthora* *cinnamomi* inoculum

*Castanea sativa* seeds were sterilized, germinated in sterile vermiculite, and then grown in a greenhouse until their roots reached a length of 5–6 cm. The seeds were then infected with *Phytophthora cinnamomi* mycelium grown in V8 medium. The infected *C. sativa* was grown in vitro at room temperature in sterile vermiculite flasks for 12, 24, and 36 h. After the incubation period, the roots were examined for the presence and extent of necrosis, and 100 mg of mycelium was collected for RNA extraction.

Twelve hours after inoculation, induced root necrosis was clearly more extensive compared to the uninfected chestnut root control. After 36 h, the lesions were quite extensive, with the root tissue being completely necrotic (Fig. 2).

This morphological analysis confirms the intensity of the interaction between *Phytophthora cinnamomi* and *Castanea sativa* in the early stages of infection. Several Nep1-like proteins, which induce necrosis, are expressed in the advanced stages of infection, indicating that these proteins contribute to the necrotrophic phase of pathogenesis. The role of the protein expressed by the *NPP1* gene in the infection and development of necrosis in chestnut trees was previously verified and explained by Martins et al. [30].

### Morphological analysis of chestnut root infection with wild type/recombinant *Phytophthoracinnamomi* inoculum

Eighteen *Castanea sativa* seeds were sterilized and germinated in sterile vermiculite. They were then grown in a greenhouse until their root length reached approximately 30 cm. Twelve of these roots were infected with mutant mycelium of *Phytophthora cinnamomi* containing the *senseIantisNPP1* silencer cassette, and another twelve were infected with mycelium of wild type *P. cinnamomi*. The infected chestnut roots were grown in triplicate at room temperature in sterile vermiculite flasks for 24, 48, and 72 h. After each period, the plants were morphologically analyzed to assess the presence and extent of necrosis, and 100 mg of mycelium was collected for RNA extraction.

A clear contrast was observed between chestnut trees infected with mutant and wild type *P. cinnamomi*. After 72 h of infection with the mutant oomycetes (carrying the *senseIantisNPP1* silencing cassette), a lower percentage of necrosis was observed in the plants compared to those infected with wild type strains, which resulted in a higher percentage of necrosis. Since the *NPP1* gene is responsible for inducing necrosis in chestnut, this result suggests the effectiveness of the *senseIantisNPP1* silencing cassette in *P. cinnamomi* transformants.

To obtain a *P. cinnamomi* strain silenced for the *NPP1* gene, a silencing cassette for the gene of interest was successfully constructed based on hairpin-RNA expression systems in the pUC57Kan vector. The silencer cassette was then cloned into the pTH210 transformation vector, and transformation was achieved using two methods: PEG-based transformation using protoplasts and transformation by electroporation using zoospores. Hygromycin B-resistant transformants were obtained using both methods.

Of the ten mycelium growths subjected to the PEG transformation procedure, only two grew on hygromycin B (200 µg/mL). These two transformants were termed linear T1 and linear T4 because they were transformed with the plasmid linear recombinant pTH210. However, after subculturing in medium containing hygromycin B, only linear T1 remained stable and survived. Of the twelve growths made from mycelium subjected to the transformation procedure by means of electroporation, all of them grew in hygromycin B. Of these transformations, six were carried out with linear recombinant pTH210 plasmid and another six with the circular recombinant plasmid. However, after subculturing in medium containing hygromycin B, only six growths were stable and survived. These were called linear TE.1, circular TE.8, circular TE.9, circular TE.10, circular TE.11, and circular TE.12. With 100% growth of hygromycin B-resistant transformants, compared to only 20% growth of selector antibiotic-resistant transformants. It was also observed that the circular recombinant plasmid was more stable and had a higher survival rate than the linear recombinant plasmid when subcultured to new media containing hygromycin selector antibiotic B (200 ug/mg).

DNA was later extracted from the transformants, and fragments belonging to the recombinant plasmid (*senseIantisNPP1*, hygromycin gene) were amplified using the PCR technique. These fragments were identified with the aid of enzymatic digestions and automatic sequencing, effectively confirming the transformation of *P. cinnamomi* with the recombinant plasmid carrying the silencer cassette for the *NPP1* gene. We also performed an analysis of protein expression using SDS-PAGE to confirm the effectiveness of the silencer cassette. Partial expression was found for the strain transformed with the linear T1 plasmid, indicating a substantial reduction in protein production compared to the wild type. The absence of expression was successfully verified for the circular TE.8 transformed strain, indicating the silencing of NPP1 protein expression.

To compare chestnut trees infected with mutant and wild type *P. cinnamomi*, we infected chestnut roots with inoculum for different times and performed a morphological analysis. A clear contrast was observed between chestnut trees infected by mutant and normal *P. cinnamomi*. After 72 h of infection with the mutant oomycetes carrying the *senseIantisNPP1* silencing cassette, a lower percentage of necrosis was observed in the plants, while a higher percentage of necrosis was observed in plants infected with wild type strains. As the *NPP1* gene is known to induce necrosis in chestnut, this result suggests the effectiveness of the *senseIantisNPP1* silencing cassette in the *P. cinnamomi* transformant.

## Concluding remarks

In conclusion, this study has demonstrated the possibility of obtaining relatively stable transforming strains with reduced or absent NPP1 production compared to the wild isolates that originated them. This is considered an important tool for achieving the general objective of this work, which aimed to contribute to the elucidation of the biological role of effector proteins in the infectious process of *Phytophthora cinnamomi*.

As future perspectives, we intend to analyze the effectiveness of the silencer cassette through RT-PCR and Next-Generation Sequencing (NGS). We believe that this will allow for more detailed functional analysis of the infection process and elucidation of the mechanisms of molecular communication between the host-pathogen during infection.

Furthermore, additional research could be performed, such as (1) transforming *Phytophthora cinnamomi* with a vector harboring multiple silencing cassettes for different genes involved in the mechanism of infection and analyzing the effect on the plant phenotype, the expression level of genes in *P. cinnamomi*, and the plant proteome; and (2) examining the interaction between *P. cinnamomi* and chestnuts by monitoring proteins tagged with different fluorescent vectors.

## Supplementary information

Below is the link to the electronic supplementary material.

Supplementary material 1 (DOCX 837.4 kb)

## Data Availability

All data generated or analyzed during this study are included in this published article (and its supplementary information files).
