# Supplementary material for: Use of iRNA in the post-transcriptional gene silencing of necrosis-inducing Phytophthora protein 1(NPP1) in Phytophthora cinnamomi
Source: Mol Biol Rep. 2023 Jun 16;50(8):6493–504. doi: 10.1007/s11033-023-08562-7 (PMC10374718; doi:10.1007/s11033-023-08562-7)
Supplement: Supplementary file 1 — Supplementary material 1 (DOCX 837.4 kb) [file 11033_2023_8562_MOESM1_ESM.docx]

**Supplementary material**

**Use of iRNA in the post-transcriptional gene silencing of *necrosis-inducing Phytophthor*a *protein 1* (NPP1) in *Phytophthora cinnamomi***

# More information about:

# MATERIALS AND METHODS

- 1. **Expression vectors**

**pUC57kan Vector**

pUC57kan is a cloning vector widely used in *Escherichia coli*. It has a size of 2710 bp and contains the kanamycin resistance gene. This plasmid was used to clone the *senseIantisNPP1* silencing cassette in *Escherichia coli.*

**pTH210 vector**

This expression vector was built by Howard S. Judelson. It has a size of 5030 bp, containing the hsp 70 promoter and the hygromycin resistance gene. This plasmid was used to insert the *senseIantisNPP1* silencer cassette into the *Phytophthora cinnamomi* genome.

**
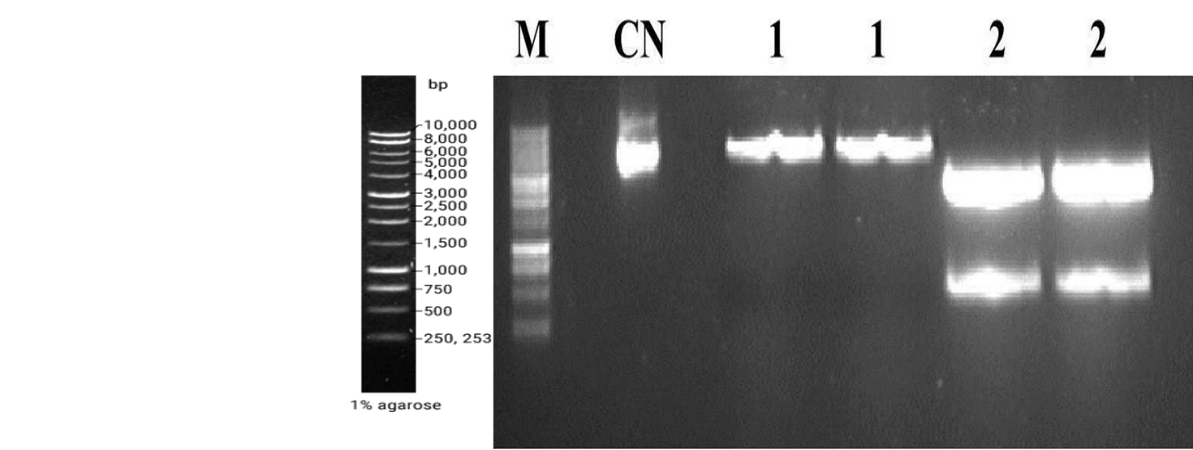
**

**Fig. 1** Enzymatic digestion of pTH210 and pUC57Kan with *Apa*I. Agarose gel 1% (w/v) in 1X TAE. M) 1kb molecular weight marker (Promega®); CN Negative control for supercoiled pTH210 plasmid DNA; 1. Plasmid pTH210 linearized by the enzyme *Apa*I; 2. Recombinant pUC57kan plasmid digested with *Apa*I resulting in two fragments.


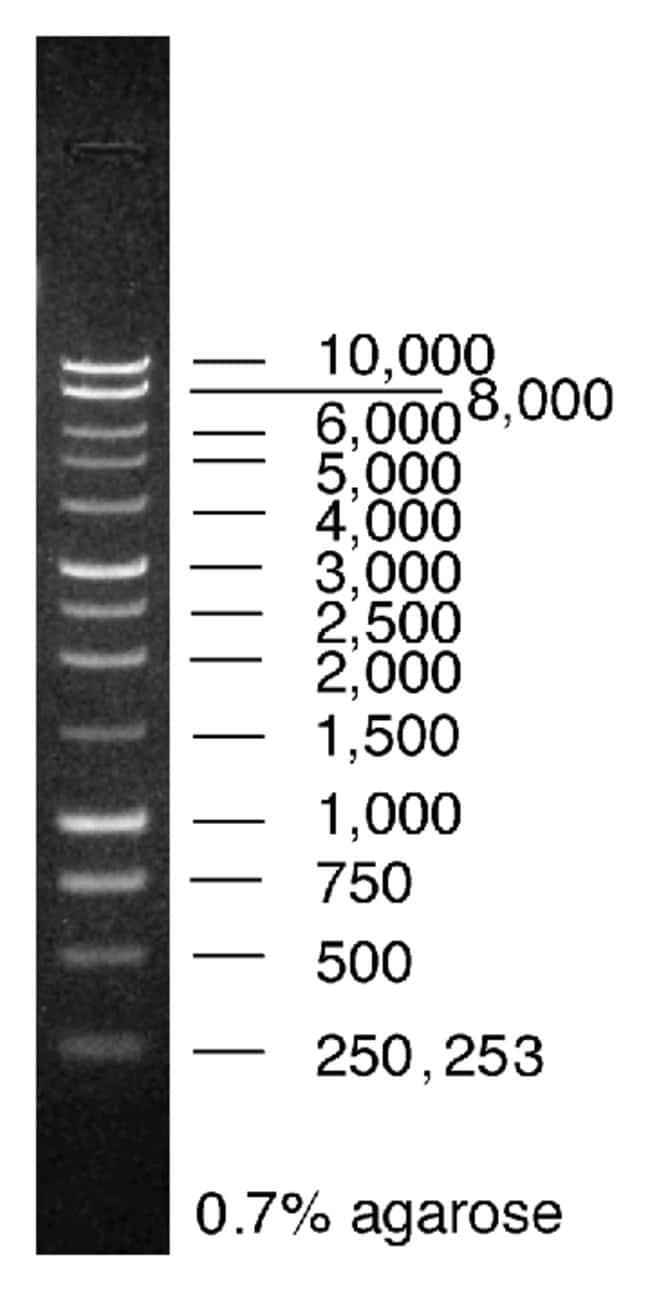

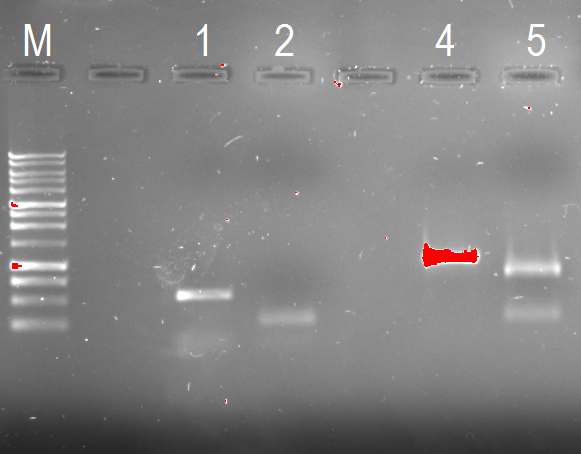


**Fig. 2** PCR products and enzymatic digestions. M. 1kb molecular weight marker (Promega®); 1. Fragment 565 bp Cassette-Hygromycin PCR Product; 2. *Nco*I digestion of the cassette-hygromycin fragment yielding two overlapping bands of size 282 bp and 283 bp. 4. 1090 bp fragment. 5. Digestion with *Pst*I of the hygromycin fragment yielding two bands, a smaller 282 bp and a larger 808 bp.

**Discussion and Results**

## **Synthesis and preparation of the *NPP1* silencing cassette**


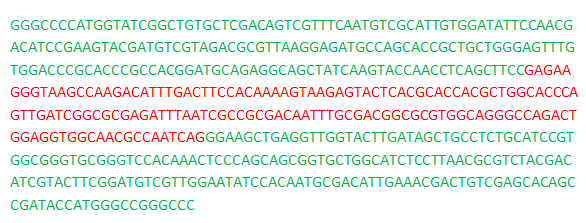


**Fig. 3** The sense, loop and antisense of the *NPP1* silencing cassette. 519 bp senseIantisNPP1 cassette sequence. In green is the sense and anti-sense sequence. In red is the intron.


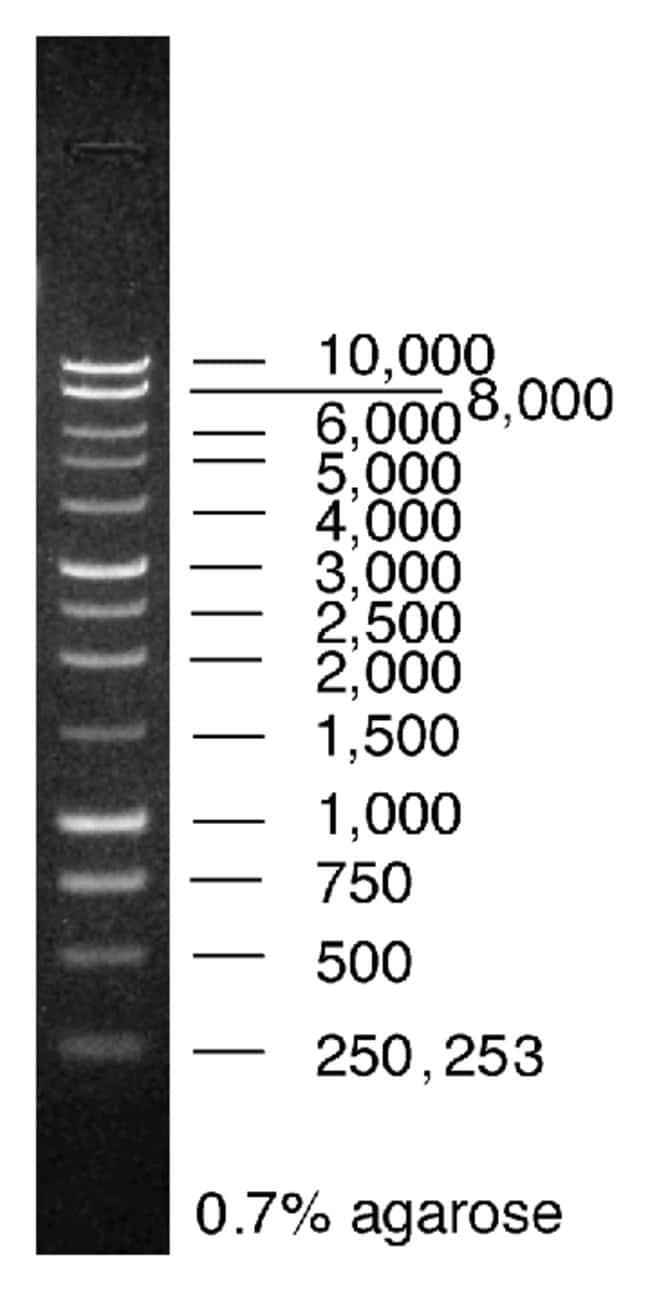

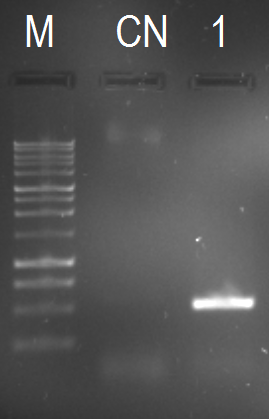


**Fig. 4** Amplification of the senseIantisNPP1 cassette segment plus hygromycin segment. M. 1kb molecular weight marker (Promega®); CN: Negative control; 1. 565 bp cassette-hygromycin PCR product.


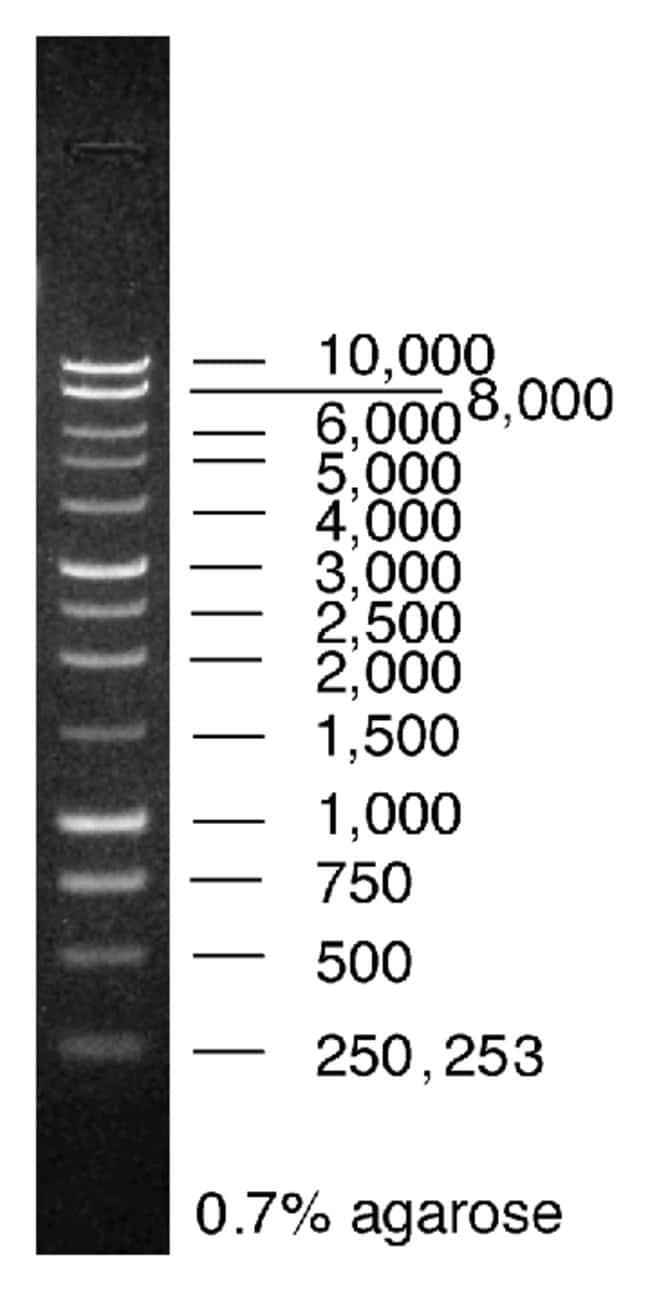

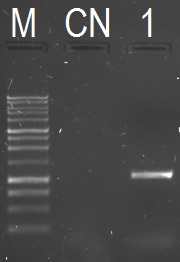


**Fig. 5** Hygromycin segment amplification. M. 1kb molecular weight marker (Promega®); CN: Negative control; 1. Hygromycin PCR product 1090 bp.


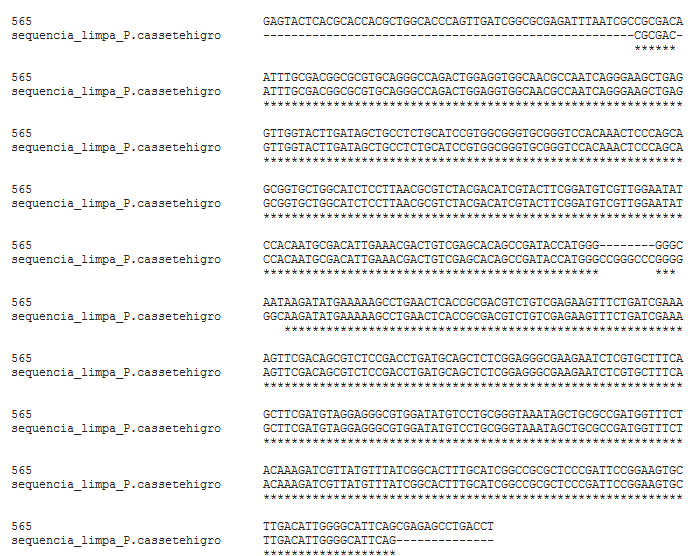


**Fig. 6** Homology between sequencing result and PCR product (Hygromycin Cassette) with evident *Apa*I (GGGCCC) restriction site.


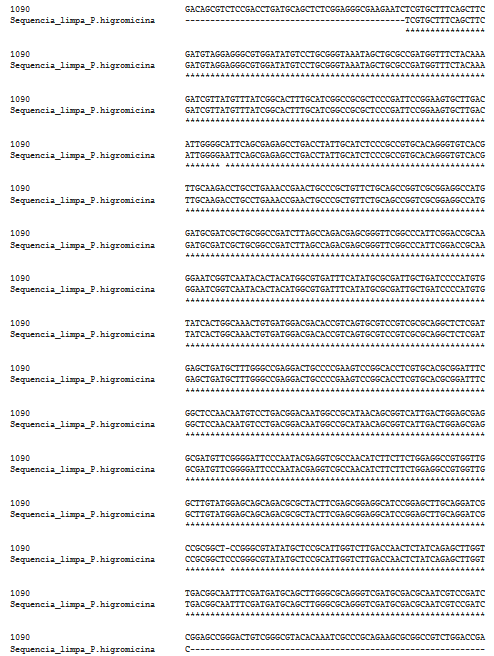


**Fig. 7** Homology between sequencing result and hygromycin PCR product.
